# Prevalence of asthma and other allergic conditions in adults in Khuzestan, southwest Iran, 2018

**DOI:** 10.1186/s12889-019-6491-0

**Published:** 2019-03-13

**Authors:** Esmaeil Idani, Hanieh Raji, Farzan Madadizadeh, Bahman Cheraghian, Maryam Haddadzadeh Shoshtari, Maryam Dastoorpoor

**Affiliations:** 1 Air Pollution and Respiratory Diseases Research Center, Ahvaz Jundishapur University of Medical Sciences, Ahvaz, Iran; 2 Research Center of Prevention and Epidemiology of Non-Communicable Disease, Department of Biostatistics and Epidemiology, School of Public Health, Shahid Sadoughi University of Medical Sciences, Yazd, Iran; 3 Department of Epidemiology and Biostatistics, School of Public Health, Tehran University of Medical Sciences, Tehran, Iran; 4 Department of Epidemiology and Biostatistics, School of Public Health, Ahvaz Jundishapur University of Medical Sciences, Ahvaz, Iran; 5 Department of Epidemiology and Biostatistics, Air Pollution and Respiratory Diseases Research Center, Ahvaz Jundishapur University of Medical Sciences, Ahvaz, Iran

**Keywords:** Prevalence, Asthma, Rhinitis, Allergic, Eczema, Adults

## Abstract

**Background:**

Health information on the dimensions of asthma and allergic conditions in Khuzestan Province, as a major industrial and polluted area in Iran as and the Middle East, is inadequate. This study was performed to measure the prevalence of asthma and other allergic conditions in adults in Khuzestan Province.

**Methods:**

This population-based cross-sectional study was carried out in 17 villages and 27 cities of Khuzestan Province during the years 2017–2018 on 20 to 65 year old respondents. Two-stage cluster sampling was used. The ECRHS (European Community Respiratory Health Survey) questionnaire was completed for individuals with additional questions regarding other allergic conditions.

**Results:**

In the study, 5720 questionnaires were distributed of which 5708 were returned. The prevalence of current asthma was 8.5% and that of asthma-like symptoms was 19.0%. The most common symptoms of asthma were nocturnal cough (13.6%), chest tightness (12.3%) and wheezing (13.1%). The prevalence of allergic rhinitis (AR), eczema and airway hyperresponsiveness were 27.2, 10.7, and 38.7%, respectively. The prevalence of current asthma was strongly correlated with age, current location (city, village), and the smoking status of respondents (*p* < 0.05).

**Conclusion:**

The prevalence of current asthma and asthma-like symptoms in Khuzestan Province is almost twice as high as in Iran. Given the high prevalence of symptoms of airway hyperresponsiveness in the entire province, it is necessary to take environmental measures to mitigate the emergence of new cases of asthma among the residents. In addition, surveillance studies are necessary to monitor the trends in the prevalence of asthma in this province.

## Background

Asthma is a chronic inflammatory disease of the respiratory tract characterized by unstable obstruction of the airflow and severe response of the airways [1]. The most common symptoms of asthma are frequent wheezing, rapid breathing, chest tightness and coughing, which usually aggravate in the presence of certain factors such as dust, activity, cigarette and air pollution [2].

Various studies have revealed that the prevalence of asthma is on the increase worldwide, especially in industrialized countries. For example, the number of persons with self-reported asthma in the United States has more than doubled between 1980 (6.5 million) and 1996 (14.5 million) [3]. It is also estimated that more than 330 million people in the world suffer from asthma, and by 2025 a hundred million more are probably added to the number of people with asthma. [4].

According to the Global Asthma Network’s 2016 study, the burden of disease attributed to asthma based on the Disability Adjusted Life Years (DALYs) index was estimated to be 23.7 million DALYs in all age groups, 56% of which were Years of Life Lost due to premature death (YLD) and 44% were Years of Life lived with Disability (YLL). In general, asthma ranks 28th among the world’s leading burden of disease [5].

The results of the studies show that the prevalence of wheezing in different countries of the world varies from 1.6 to 36.8%. It is less than 5% in Albania, China, Greece, Indonesia, Romania and Russia. In countries like Australia, New Zealand, Ireland and the United Kingdom, it ranges from 29 to 32%. Australia, for example, has reported wheezing as 29.4%, Austria 11.9%, Ireland 15.2%, Brazilian boys 7.3%, and girls 4.9% [6].

There are few studies on the prevalence of asthma in adults in the eastern Mediterranean region classified by the World Health Organization, especially in Iran [7, 8]. In a cross-sectional study in Iran in 2015, the rate of current asthma and asthma-like symptoms in adults was 4.7 and 8.9, respectively [9].

Asthma is a multifactorial disease in which family as well as infectious, allergic, socioeconomic, psychological and environmental factors play a role [10]. Environmental factors such as exposure to various allergens, irritants, and industrial pollutants and particulate matter are involved in developing countries [11, 12]. For developed countries, outdoor pollutants such as benzene, particulate matter and irritant gases including nitrogen dioxide (NO_2_), ozone (O_3_), sulfur dioxide (SO_2_) increase the incidence of respiratory diseases, especially asthma [13, 14].

The Province of Khuzestan has been exposed to various pollutants for many years, the most important sources of which are micro-waste, industrial and non-industrial pollutants. Ahvaz is the capital of Khuzestan Province. The rate of pollution in this city is increasing and becoming more severe every day [15, 16]. According to the World Health Organization’s report in 2013, the City of Ahwaz, in terms of the average annual amount of suspended particles of less than 10 μm (372 μg/m^3^), is the most polluted city in the world thus far [17].

Among the requirements of this study was the occurrence of multiple respiratory outbreaks during three years from 2013 to 2015 as the result of the first autumn rainfall in the Khuzestan Province, which seems to be influencing the occurrence of asthma and allergic conditions [18]. Also, in the study of Fazlollahi et al. in Iran in 2015, the Khuzestan Province ranks the highest as regards the incidence of current asthma [9].Therefore, conducting an accurate study on the epidemiology of asthma and allergic conditions in the province seemed necessary. Therefore, this study was carried out to estimate the prevalence of asthma and other allergic conditions in adults in the Khuzestan Province.

## Methods

### Study area

The study was conducted in the Khuzestan Province, southwest of Iran, located at the geographical coordinates of 31.33 ° N and 48.69 ° E. The Khuzestan Province shares border with Iraq and the Persian Gulf, which is the center of oil and gas production in Iranian [19]. The area of the Khuzestan Province is 64,057 km^2^ and, with a population of 4,710,506 people, it is considered as the fifth-largest populated region of Iran. The capital of the Khuzestan Province is Ahvaz City [19].

### Study design

This cross-sectional study was conducted during September 2017 to February 2018 on the adult population of the Khuzestan Province. The study was carried out in 24 counties of Khuzestan through sampling from 27 cities and 17 villages. The inclusion criteria were male or female from 20 to 65 years of age and giving personal consent for participation in the study. Exclusion criteria were individuals with specific illnesses, disabilities, and/or a mental state, and dementia at the time of study that prevented them from co-operating.

### Sample size

According to a similar study by Mehrabi et al., which estimated the prevalence of asthma in adults aged 15–65 years in Kurdistan to be 0.023 [20] and the National Census of 2016 which reports the population of the Khuzestan Province as 4,710,509 people, and the following formula, this research considered a sample size of 2600 people. Drawing upon the effect size 2, the sample size was obtained as 5200 respondents. Taking into account that approximately 10% of the respondents might not respond, we calculated the final sample size as 5720.

$$ {\displaystyle \begin{array}{l}n=\frac{Nz_{1-\frac{\alpha }{2}}^2p\left(1-p\right)}{d^2\left(N-1\right)+{z}_{1-\frac{\alpha }{2}}^2p\left(1-p\right)},\mathrm{N}=4710509,\alpha =0.05,{z}_{1-\frac{\alpha }{2}}=1.96,p=0.023,d=0.0054\Rightarrow n\simeq 2600\\ {}\end{array}} $$

### Sampling frame and sampling strategy

In this study, two-stage cluster sampling was used. In the first stage, all clusters in Khuzestan Province were selected. In the second stage, 143 clusters were selected by random systematic sampling from the whole cluster, taking into account the probability proportional to their size. The number of clusters in urban and rural areas was selected based on their population’s proportion to the population of Khuzestan Province. The list of census enumeration areas, population and geographic map of each cluster were prepared through correspondence with the Iranian Statistics Center and the latest census data (2017). At the end, 143 clusters each with a cluster size of 40 persons were selected. Each cluster included 8 men and 8 women aged 20 to 29 years old, 8 men and 8 women in the range of 30–44 years old, and 4 men and 4 women aged 45–65 years.

After receiving the necessary trainings, enumerators began sampling. They were handed maps of selected clusters and referred to the address of each cluster from the southwest point of that cluster in the clockwise direction. For each cluster, sampling was carried out until 40 respondents agreed to participate.

Of each family, not more than one person was selected for any age group, and if there were more than one person in a family for a certain age group, one was chosen among those whose day and month of birth were closer to the day of the survey. Questioning was done in the afternoons and holidays with the consent of the respondents.

### Questionnaire

The instrument for studying the prevalence of asthma and asthma symptoms was the ECRHS questionnaire, [21] which was previously used in domestic studies of standardization [7] and localization. The ECRHS questionnaire is the standard tool used in the survey of “the European Respiratory Society” in 48 centers in 17 European countries and 5 non-European countries between 1990 and 1995 in adults aged 20–44 [21].

In the ECRHS, asthma symptoms include: Wheezing in the past 12 months, wheezing with shortness of breath in the last 12 months, wheezing in the absence of cold during the past 12 months, feeling tightness in the chest while waking up in the past 12 months, nocturnal cough and dyspnea attacks in the last 12 months, a history of asthma attack in the last 12 months, recent anti-asthma medication, and nasal allergies.

Based on the ECRHS questionnaire, current asthma is defined as recently taking anti-asthma medication or having an asthma attack in the last 12 months. People with asthma-like symptoms are those individuals who have at least one symptom of asthma including wheezing, waking up with a feeling of tightness in the chest, waking up with dyspnea and coughing attacks in the last 12 months [21].

The questionnaire included questions about age, gender, education level, marital status, employment status, active smoking, passive smoking, quantity of cigarettes smoked, duration of smoking, physician-confirmed asthma (Do you have asthma (confirmed by doctor)?), allergic rhinitis, airway hyperresponsiveness and eczema condition.

In relation to the measurement of the prevalence of “airway hyperresponsiveness”, a multi-choice question was asked: Which of the following irritates you and starts symptoms such as shortness of breath, cough, sneezing, runny nose, itching of the eyes, red eye and the hives? pollen, dried plants, cat, dog, bird, cosmetics, spray, colognes, insecticides, rubber gloves, metal and jewelry, home dust, air pollution, exercise, nervousness, cold weather, humidity, cool air from a cooler, sweeping and any other substance (food, medicine and plant) that the person expresses.

*Asthma* is an inflammatory disease affecting the airways of the lungs and leaving long-term impacts on the patient [22]. The characteristics of the disease include: recurring symptoms, reversible airflow obstruction, and bronchospasm. The symptoms of this disease include wheezing, coughing, chest tightness, and shortness of breath [23].

*Rhinitis* is referred to inflammation and irritation of the mucous membrane which is inside the nose. The typical symptoms of rhinitis are a stuffy and runny nose and sneezing. This inflammation type is the result of exposure to bacteria, viruses, irritants or allergens [24].

*Airway hyperresponsiveness* is an increased sensitivity of the airways to a constrictor or agonist inhaled by the patient, a steeper slope of the dose-response curve, and a bigger maximal reaction to the agonist [25].

*Eczema* is referred to a group of ailments that leads to skin inflammation. The characteristics of these ailments are itching, red skin and a rash [26].

### Statistical analysis

Considering that the results were obtained for 24 countries and were to be generalized to the total population of 20–65 years living in Khuzestan Province, all cities and villages were given weights. This means that each sample represents several people.

The total weight is obtained from the inverse multiplication of two weights: one is the weight associated with the cluster selection, and the other is the weight of each sample from the clusters. Total weight was obtained using a formula. In the formula, N_p_ is the total number of clusters in Khuzestan Province and n_p_ is the number of selected clusters (143 clusters). N_c_ and n_c_ are the total population of 20 to 65 year olds and the sample size in each cluster, respectively.

$$ PWT=\left(\frac{N_p}{n_p}\right)\times \left(\frac{N_c}{n_c}\right) $$

To estimate the descriptive results, frequency, mean and standard deviation were used. To measure the analytical results and examine the relationship between the dependent variables (asthma symptoms and allergic conditions) and the independent variables (age, sex, educational status, residence, and smoking status), the chi-square test and multiple logistic regression analysis (ENTER method) were used. An analysis was done using Stata 10, and a *p*-value below 0.05 was considered significant.

## Results

### Distribution of the respondents according to demographic characteristics

In the study, 5720 questionnaires were distributed of which 5708 were returned. Some of the variables such as education level and active smoking were non-response. The mean ± SD age of the respondents in the study was 34.5 ± 11.8 years (95% CI: 34.2–34.8). The proportion of men was 50.7% and women were 49.3%. The majority of the respondents held high school and associate’s degrees (37.9%), were married (62.0%), lived in urban areas (87.4%) and were employed (53.2%). The prevalence of smoking in the age group of 20 to 65 years was 15.7%. The average smoking rate was 18.1 ± 9.3 (95% CI: 16.6–19.7) cigarettes a day and the mean smoking duration was 14.1 ± 9.0 years (95% CI: 12.8–15.4). More details about the demographics of the respondents are shown in Table 1.

**Table 1:** Distribution of Respondents According to Demographic Characteristics in Khuzestan^b^

Variable	Class	N	Weighted %	95% CI	
Gender	Male	2882	50.7	49.8	51.6
	Female	2826	49.3	48.4	50.2
Age	20–29 years	2262	39.6	39.0	40.2
	30–44 years	2256	39.4	38.8	40.0
	45–65 years	1190	21.0	20.2	21.8
Education level ^a^	Illiterate	313	5.7	4.6	6.8
	Low	1585	29.1	26.4	31.8
	Moderate	2056	37.9	36.2	39.7
	High	1460	27.2	24.0	30.4
Marital status	Married	3487	62.0	60.2	63.9
	Single	2077	36.9	35.0	38.8
	Divorced	19	0.4	0.2	0.6
	Widow/widower	41	0.7	0.5	1.0
Region	Urban	5013	87.4	81.9	92.9
	Rural	695	12.6	7.1	18.1
Employment status	Employed	1483	53.2	50.6	55.7
	Unemployed	1327	46.8	44.3	49.4
Active smokers	Yes	882	15.7	13.5	17.9
	No	4775	84.3	82.1	86.5
Passive smokers	Yes	3292	58.1	52.9	63.4
	No	2363	41.9	36.6	47.1

^a^: Low educational level (primary school & middle school), moderate educational level (high school and associated degree), and high educational level (bachelor, master and doctorate degree)

^b^: n may not total to 5708 in some variables due to non-response

### Prevalence of asthma symptoms and other allergic conditions

In the study, the prevalence of current asthma was 8.5% and asthma-like symptoms were 19%. The most common symptoms of asthma were nocturnal cough (13.6%), chest tightness (12.3%) and wheezing (13.1%). The prevalence of AR, eczema and airway hyperresponsiveness was 27.2, 10.7, and 38.7%, respectively (Table 2).

**Table 2:** Prevalence of Asthma Symptoms and Other Allergic Conditions in Overall and According to Gender

Asthma symptoms and other allergic conditions	Total			Gender				
				Male		Female		*P*-value^¥^
	N^b^	Yes	%^a^ (95% CI)	Yes	%^a^(95% CI)	Yes	%^a^(95% CI)	
Asthma-like symptoms	5659	1059	19.0(15.9–22.1)	540	19.2(15.6–22.5)	519	18.7(15.7–22.0)	0.839
Wheezing	5655	728	13.1(10.8–15.4)	374	13.2(10.7–15.8)	354	13.0(10.5–15.4)	0.781
Wheezing with shortness of breath	5539	674	12.3(10.3–14.2)	348	12.5(10.2–14.8)	326	12.0(9.9–14.1)	0.624
Wheezing in the absence of a cold	5516	656	12.1(10.0–14.1)	323	11.8(9.4–14.0)	333	12.3(10.2–14.5)	0.468
Waking with tightness in the chest	5660	690	12.3(10.2–14.3)	349	12.2(9.9–14.6)	341	12.3(10.1–14.5)	0.947
Woken by an attack of breathlessness	5658	580	10.3(8.6–12.0)	303	10.6(8.5–12.7)	277	10.0(8.2–11.7)	0.512
Woken by attack of cough	5668	768	13.6(11.4–15.8)	380	13.3(10.8–15.8)	388	13.8(11.6–16.2)	0.473
Current asthma	5672	478	8.5(7.0–10.0)	231	8.1(6.6–9.7)	247	8.9(7.0–10.7)	0.278
Asthma attack	5646	410	7.3(5.8–8.7)	195	6.8(5.4–8.2)	215	7.7(6.0–9.5)	0.174
Medications for asthma	5666	417	7.4(6.1–8.8)	198	7.0(5.6–8.4)	219	7.9(6.2–9.5)	0.192
Physician-confirmed asthma	5658	359	6.4(5.3–7.5)	171	6.0(4.8–7.2)	188	6.8(5.4–8.1)	0.245
Allergic Rhinitis	5671	1544	27.2(25.1–29.4)	694	24.2(22.0–26.5)	850	30.4(27.8–32.9)	< 0.001*
Airway hyperresponsiveness	5708	2190	38.7(35.0–42.3)	1000	35.1(31.3–38.9)	1190	42.3(38.4–46.3)	< 0.001*
Eczema	5663	605	10.7(9.2–12.3)	277	9.7(8.0–11.5)	328	11.8(10.0–13.5)	0.021*

^a^: Prevalence estimates were weighted using normalized cross-sectional weights

^**b**^: n may not total to 5708 in some variables due to non-response

¥ χ^2^ test

*P < 0.05

The results of bivariate analysis showed that the prevalence of current asthma, asthma-like symptoms, and all signs of asthma include: wheezing, wheezing with dyspnea, wheezing in the absence of colds, chest tightness, nocturnal dyspnea, nocturnal cough, asthma attack, medications for asthma and physician-confirmed asthma were not different in men and women (*P*-value> 0.05). However, AR, airway hyperresponsiveness and eczema were more prevalent in women (P-value< 0.05) (Table 2).

The prevalence of current asthma, asthma-like symptoms, all signs of asthma and physician-confirmed asthma in the age group of 45 to 65 years were significantly higher compared to other age groups (*P*-value< 0.05). Meanwhile, AR were more prevalent in young people aged 20–29 years (*P*-value < 0.05) (Table 3).

**Table 3:** Prevalence of Asthma Symptoms and Other Allergic Conditions According to Age

Asthma symptoms and other allergic conditions	Age (years)						
	20–29		30–44		45–65		*P*-value^¥^
	Yes	%^a^ (95% CI)	Yes	%^a^ (95% CI)	Yes	%^a^ (95% CI)	
Asthma-like symptoms	353	15.8(12.7–18.9)	397	18.0(14.6–21.3)	309	26.8(22.5–31.2)	< 0.001*
Wheezing	247	11.1(8.6-13.5)	273	12.4(9.8–15.0)	208	18.2(14.9–21.6)	< 0.001*
Wheezing with shortness of breath	223	10.1(8.1–12.2)	259	11.9(9.6–14.3)	192	16.9(14.1–19.7)	< 0.001*
Wheezing in the absence of a cold	211	9.7(7.8–11.7)	241	11.2(8.9–13.5)	204	18.0(14.7–21.4)	< 0.001*
Waking with tightness in the chest	218	9.7(7.6–11.8)	269	12.2(9.7–14.6)	203	17.3(14.3–20.3)	< 0.001*
Woken by an attack of breathlessness	191	8.5(6.7–10.3)	211	9.5(7.5–11.5)	178	15.2(12.4–18.0)	< 0.001*
Woken by attack of cough	250	11.1(8.9–13.4)	289	12.9(10.3–15.6)	229	19.5(16.3–22.8)	< 0.001*
Current asthma	162	7.2(5.6–8.9)	185	8.3(6.5–10.1)	131	11.3(9.0–13.7)	< 0.001*
Asthma attack	139	6.2(4.7–7.8)	158	7.0(5.3–8.8)	113	9.7(7.6–11.8)	0.001*
Medications for asthma	139	6.2(4.8–7.6)	158	7.1(5.4–8.7)	120	10.4(8.3–12.4)	< 0.001*
Physician-confirmed asthma	122	5.5(4.1–6.9)	136	6.1(4.7–7.6)	101	8.7(7.0–10.4)	0.005*
Allergic Rhinitis	710	31.6(28.4–34.7)	553	24.7(22.2–27.2)	281	23.9(21.1–26.8)	< 0.001*
Airway hyperresponsiveness	956	42.2(38.1–46.3)	795	35.8(31.7–39.9)	439	37.3(32.7–41.9)	0.001*
Eczema	276	12.3 (10.2–14.4)	209	9.4(7.7–11.0)	120	10.3(8.0–12.6)	0.012*

^a^: Prevalence estimates were weighted using normalized cross-sectional weights

^¥^ χ^2^ test

*P < 0.05

The prevalence of airway hyperresponsiveness among people with higher education is higher than others (*P*-value < 0.05). In contrast, the prevalence of asthma-like symptoms, wheezing with dyspnea, wheezing in the absence of colds, chest tightness, nocturnal dyspnea and nocturnal cough were significantly higher among illiterate people than others (P-value< 0.05)(Table 4).

**Table 4:** Prevalence of Asthma Symptoms and Other Allergic Conditions According to Education level

Asthma symptoms and other allergic conditions	Education level ^b^								
	Illiterate		Low		Moderate		High		P-value^¥^
	Yes	%^a^(95% CI)	Yes	%^a^(95% CI)	Yes	%^a^(95% CI)	Yes	%^a^(95% CI)	
Asthma-like symptoms	72	23.8(18.3–29.3)	243	15.6(12.0–19.1)	333	16.4(13.4–19.4)	309	22.0(16.5–27.4)	0.009*
Wheezing	46	15.1(10.3-20.0)	175	11.2(8.5–14.0)	231	11.4(9.2–13.7)	219	15.7(11.2–20.1)	0.050
Wheezing with shortness of breath	49	16.8(11.9–21.6)	159	10.3(8.3–12.4)	223	11.1(9.1–13.1)	191	13.6(10.0–17.3)	0.038*
Wheezing in the absence of a cold	47	16.1(11.2–21.0)	151	9.9(7.9–12.0)	212	10.7(8.6–12.8)	179	12.9(9.8–15.9)	0.025*
Waking with tightness in the chest	54	17.8(12.6–22.9)	164	10.5(8.4–12.6)	219	10.7(8.7–12.7)	182	12.8(9.4–16.2)	0.019*
Woken by an attack of breathlessness	49	16.1(11.6–20.7)	137	8.8(7.0–10.7)	193	9.4(7.6–11.3)	146	10.2(7.5–12.8)	0.013*
Woken by attack of cough	63	20.8(15.5–26.1)	188	12.0(9.2–14.8)	251	12.3(9.9–14.7)	200	13.8(10.5–17.1)	0.017*
Current asthma	35	11.6(7.5–15.7)	124	8.0(6.3–9.7)	154	7.6(6.2–9.1)	145	9.9(5.8–14.1)	0.260
Asthma attack	30	9.9(6.1–13.7)	102	6.5(5.0–8.1)	138	6.8(5.5–8.1)	124	8.5(4.4–12.5)	0.341
Medications for asthma	31	10.2(6.5–13.9)	116	7.5(5.8–9.2)	127	6.3(5.0–7.5)	127	8.7(5.0–12.4)	0.217
Physician-confirmed asthma	28	9.3(5.6–13.0)	100	6.5(4.9–8.1)	114	5.6(4.5–6.8)	101	7.0(4.5–9.4)	0.254
Allergic Rhinitis	78	24.8(19.9–29.7)	430	27.4(24.5–30.3)	525	25.6(23.1–28.2)	428	29.7(26.2–33.2)	0.098
Airway hyperresponsiveness	108	34.0(27.6–40.4)	563	35.8(32.2–39.3)	733	35.7(32.1–39.4)	651	45.2(38.8–51.7)	0.001*
Eczema	42	13.3(9.2-17.3)	152	9.8(8.0–11.5)	187	9.1(7.4–10.8)	197	13.7(10.7–16.7)	0.001*

^a^: Prevalence estimates were weighted using normalized cross-sectional weights

^b^: Low educational level (primary school & middle school), moderate educational level (high school and associated degree), and high educational level (bachelor, master and doctorate degree)

^¥^ χ^2^ test

*P < 0.05

Current asthma, wheezing with dyspnea, wheezing in the absence of colds, nocturnal cough, asthma attacks and medications for asthma were more prevalent in urban areas compared to rural areas (P-value < 0.05).It is worth to point out that the prevalence of current asthma, asthma-like symptoms, wheezing, nocturnal cough, medications for asthma and physician-confirmed asthma were significantly higher in smokers than non-smokers (P-value < 0.05). Moreover, AR, airway hyperresponsiveness and eczema among smokers were more prevalent (P-value< 0.05) (Table 5). The results of the adjusted model confirm these findings, except for relationship between signs of asthma and education (Table 6).

**Table 5:** Prevalence of Asthma Symptoms and Other Allergic Conditions According to Region and Active smoking

Asthma symptoms and other allergic conditions	Region					Active smoking				
	Urban		Rural		P-value^¥^	Yes		NO		*P*-value^¥^
	Yes	%^a^ (95% CI)	Yes	%^a^(95% CI)		Yes	%^a^(95% CI)	Yes	%^a^(95% CI)	
Asthma-like symptoms	968	19.7 (16.3–23.2)	91	13.7(7.4–20.0)	0.133	201	23.1(18.6–27.6)	839	18.0(14.8–21.2)	0.010*
Wheezing	670	13.7 (11.2–16.3)	58	8.9(4.3–13.5)	0.110	150	17.3(13.5–21.1)	560	12.0(9.7–14.4)	0.002*
Wheezing with shortness of breath	625	13.0 (10.8–15.2)	49	7.1(4.2–10.0)	0.005*	121	14.1(11.1-17.2)	538	11.7(9.6–13.7)	0.107
Wheezing in the absence of a cold	608	12.8 (10.5–15.1)	48	7.1(4.0–10.2)	0.012*	117	13.8(10.7-17.0)	527	11.5(9.4–13.7)	0.132
Waking with tightness in the chest	630	12.8 (10.5–15.1)	60	8.9(5.3–12.5)	0.102	125	14.4(11.2–17.5)	554	11.8(9.6–14.0)	0.106
Woken by an attack of breathlessness	528	10.7 (8.8–12.6)	52	7.6(4.6–10.7)	0.129	104	12.0(9.3–14.6)	466	9.9(8.1–11.7)	0.115
Woken by attack of cough	706	14.2 (11.8–16.7)	62	9.1(5.6–12.6)	0.033*	143	16.4(13.1-19.7)	613	12.9(10.6–15.3)	0.033*
Current asthma	447	9.1 (7.4–10.8)	31	4.5(2.3–6.8)	0.008*	103	11.9(9.1-14.7)	367	7.8(6.1–9.4)	0.004*
Asthma attack	385	7.8 (6.2–9.4)	25	3.5(1.6–5.5)	0.006*	84	9.7(7.2-12.2)	321	6.8(5.2–8.4)	0.030*
Medications for asthma	387	7.9 (6.4–9.4)	30	4.4(2.2–6.5)	0.026*	85	9.8(7.4-12.3)	326	6.9(5.4–8.4)	0.026*
Physician-confirmed asthma	329	6.7 (5.5–7.9)	30	4.5(1.9–7.2)	0.204	72	8.3(6.1–10.6)	280	6.0(4.8–7.1)	0.031*
Allergic Rhinitis	1323	26.7 (24.5–29.0)	190	27.1(21.4–32.7)	0.907	286	32.8(29.0–36.7)	1209	25.5(23.3–27.7)	< 0.001*
Airway hyperresponsiveness	1913	38.3 (34.5–42.2)	277	40.8(30.9–50.7)	0.646	393	44.6(39.5–49.7)	1767	37.3(33.5–41.1)	0.006*
Eczema	510	10.3 (8.7–12.0)	95	13.5(9.8–17.2)	0.099	121	13.7(10.9–16.4)	472	10.0(8.5–11.6)	0.005*

^a^: Prevalence estimates were weighted using normalized cross-sectional weights

^¥^ χ^2^ test

*P < 0.05

**Table 6:** Multiple logistic regression analysis of risk factors for Current asthma and other allergic conditions ^¥^

Asthma symptoms and other allergic conditions	Male ^a^	Age ^b^		Education ^c^			Urban ^d^	Smokers ^e^
		30–44	45–65	Low	Moderate	High		
	OR(95% CI)	OR(95% CI)	OR(95% CI)	OR(95% CI)	OR(95% CI)	OR(95% CI)	OR(95% CI)	OR(95% CI)
Asthma-like symptoms	1.03(0.88–1.20)	1.17(0.98–1.40)	2.16(1.70–2.74)*	0.82(0.57-1.18)	1.01(0.70–1.47)	1.47(0.89–2.43)	1.43(0.79–2.61)	1.39(1.08–1.78)*
Wheezing	0.99(0.82–1.21)	1.17(0.95–1.44)	1.99(1.50–2.64)*	0.98(0.64-1.52)	1.15(0.72–1.83)	1.68(0.92–3.07)	1.42(0.76–2.64)	1.55(1.17–2.04)*
Wheezing with shortness of breath	1.04(0.86–1.27)	1.25(1.01–1.54)*	1.90(1.44-2.51)*	0.76(0.52-1.10)	0.89(0.59–1.35)	1.11(0.66–1.88)	1.78(1.16–2.75)*	1.25(0.96-1.64)
Wheezing in the absence of a cold	0.97(0.80–1.16)	1.19(0.96–1.47)	2.27(1.73–2.98)*	0.84(0.57-1.24)	1.06(0.69–1.64)	1.23(0.76–2.22)	1.69(1.02–2.82)*	1.25(0.93-1.67)
Waking with tightness in the chest	1.04(0.86–1.26)	1.30(1.05–1.61)*	2.06(1.59-2.67)*	0.74(0.51-1.10)	0.84(0.55–1.28)	1.04(0.62–1.74)	1.48(0.88–2.49)	1.24(0.94–1.65)
Woken by an attack of breathlessness	1.07(0.87–1.33)	1.12(0.89–1.40)	2.00(1.49–2.70)*	0.68(0.47-1.00)	0.82(0.54–1.22)	0.92(0.55–1.53)	1.39(0.85–2.28)	1.22(0.92–1.61)
Woken by attack of cough	0.94(0.79–1.12)	1.18(0.97–1.44)	2.01(1.58–2.56)*	0.72(0.49-1.05)	0.83(0.57–1.20)	0.95(0.60–1.48)	1.65(1.01–2.68)*	1.38(1.06-1.80)*
Current asthma	0.88(0.73–1.06)	1.19 (0.95–1.50)	1.71 (1.28–2.28)*	0.88(0.56-1.39)	0.87(0.55–1.39)	1.17(0.58–2.36)	2.08(1.21–3.56)*	1.55(1.13-2.13)*
Asthma attack	0.83(0.68–1.02)	1.17(0.91–1.50)	1.76(1.31–2.36)*	0.87(0.55-1.37)	0.95(0.59–1.52)	1.23(0.58–2.61)	2.24(1.20–4.17)*	1.41(0.99-1.99)
Medications for asthma	0.85(0.69–1.06)	1.14(0.90–1.46)	1.77(1.32–2.37)*	0.93(0.58-1.49)	0.82(0.51–1.32)	1.18(0.58–2.38)	1.80(1.05–3.10)*	1.40(1.01-1.95)*
Physician-confirmed asthma	0.86(0.68–1.07)	1.11(0.82–1.51)	1.50(1.06–2.13)*	0.82(0.50-1.36)	0.72(0.43–1.21)	0.91(0.47–1.77)	1.45(0.78–2.72)	1.31(0.95–1.81)
Allergic Rhinitis	0.72(0.63–0.81)*	0.70(0.59-0.82)*	0.66(0.53-0.82)*	1.03(0.76-1.40)	0.91(0.65–1.28)	1.13(0.78–1.63)	0.99(0.73–1.35)	1.35(1.10–1.66)*
Airway hyperresponsiveness	0.71(0.63–0.80)*	0.74(0.62-0.88)*	0.83(0.67-1.04)	1.14(0.82–1.56)	1.15(0.83–1.59)	1.74(1.15–2.64)*	0.88(0.60-1.30)	1.30(1.05–1.60)*
Eczema	0.78(0.64-0.93)*	0.70(0.57-0.88)*	0.79(0.57-1.08)	0.69(0.46–1.02)	0.66(0.43–1.03)	1.08(0.67–1.74)	0.70(0.49–0.99)*	1.33(1.03-1.71)*

^¥^ Results are from multiple logistic regression analysis with normalized cross-sectional weights (adjusted for sex, age, education, region and active smoking)

^a^: female, ^b^: 20–29 years, ^c^: Illiterate, ^d^: rural and ^e^: non-smokers is referent category

*P < 0.05

### Prevalence of asthma symptoms and other allergic conditions in different counties

**Fig. 1:**
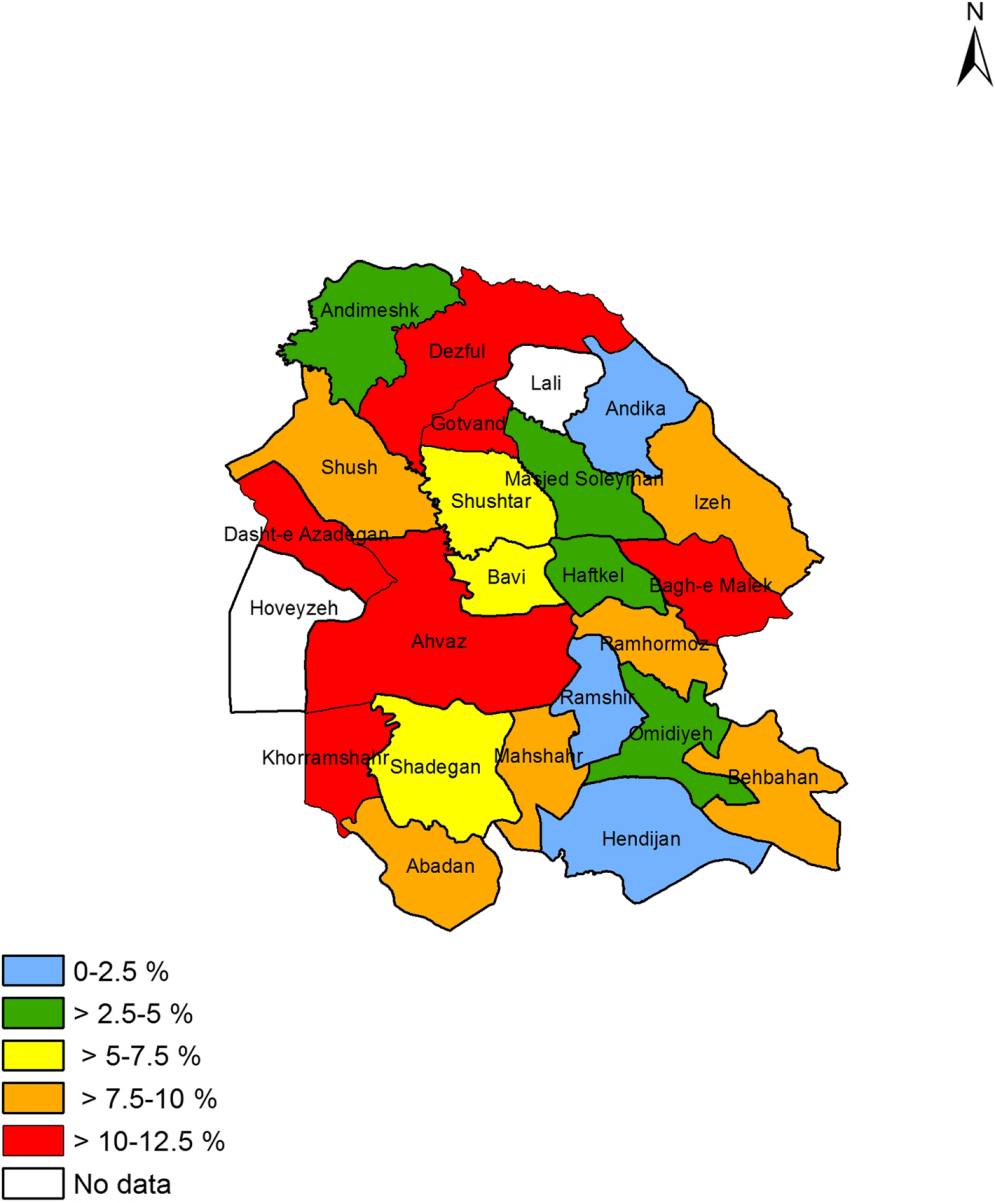
Prevalence of current asthma in Khuzestan province

**Fig. 2:**
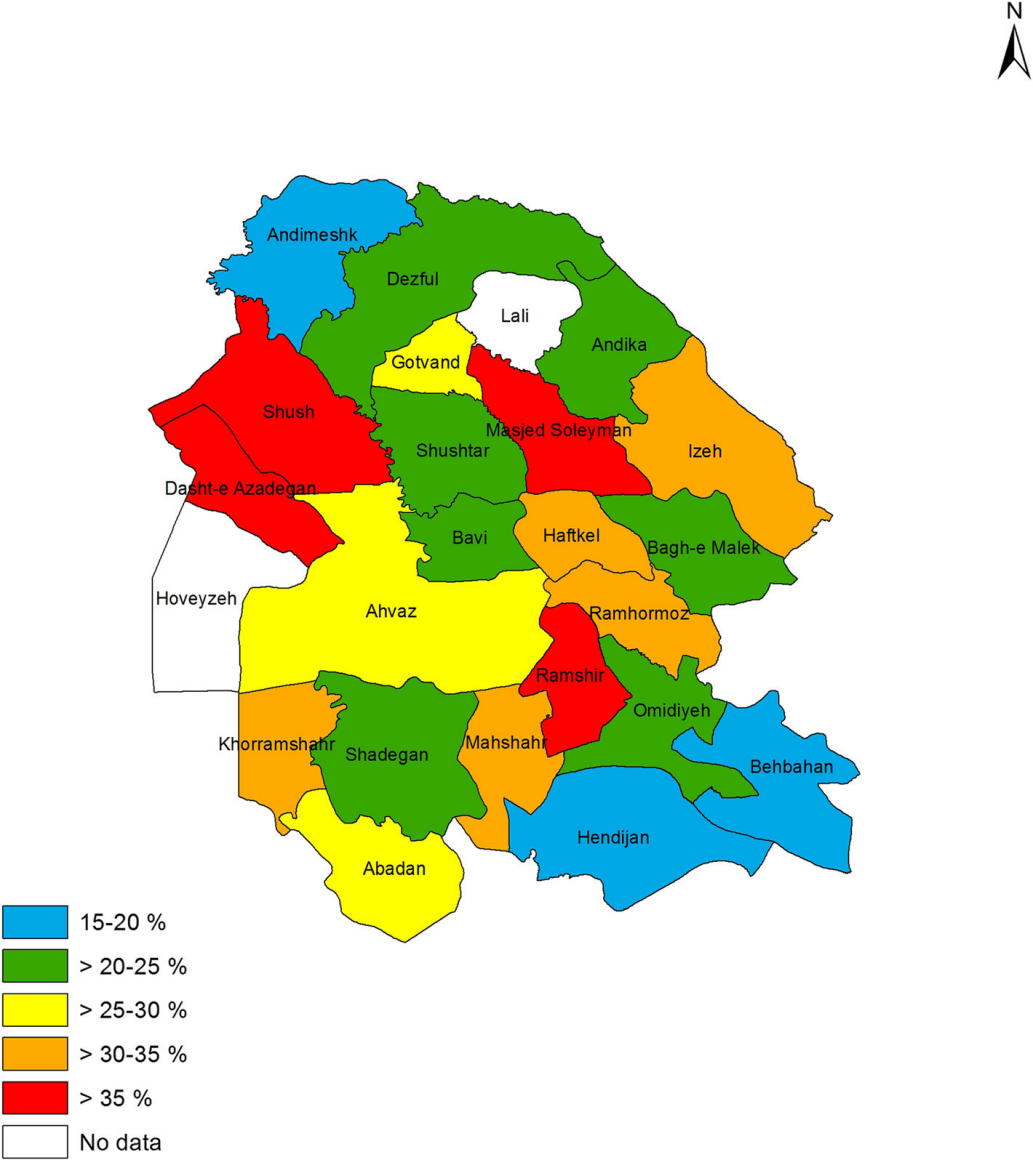
Prevalence of allergic rhinitis in Khuzestan province

**Fig. 3:**
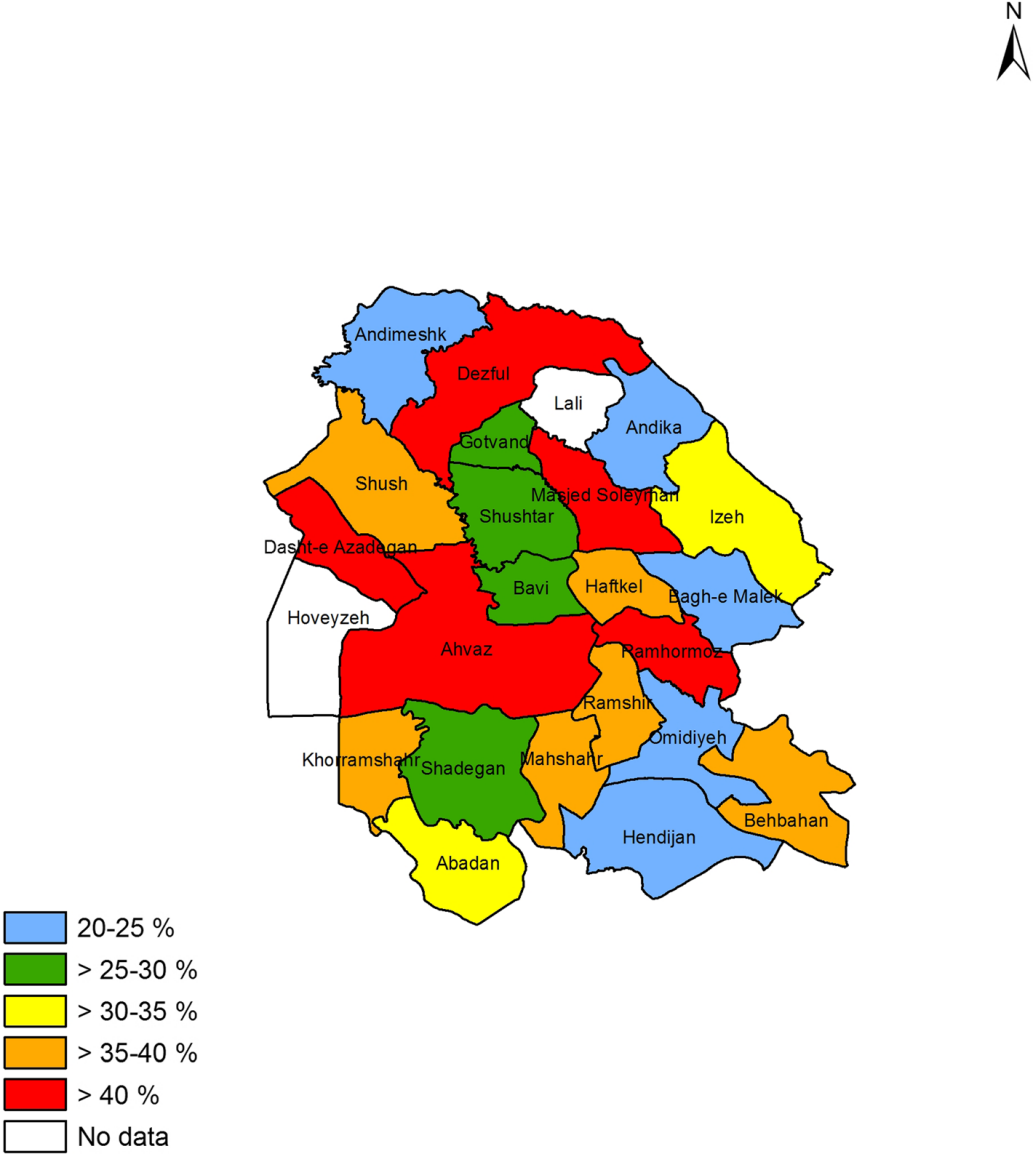
Prevalence of hyperresponsiveness in Khuzestan province

**Fig. 4:**
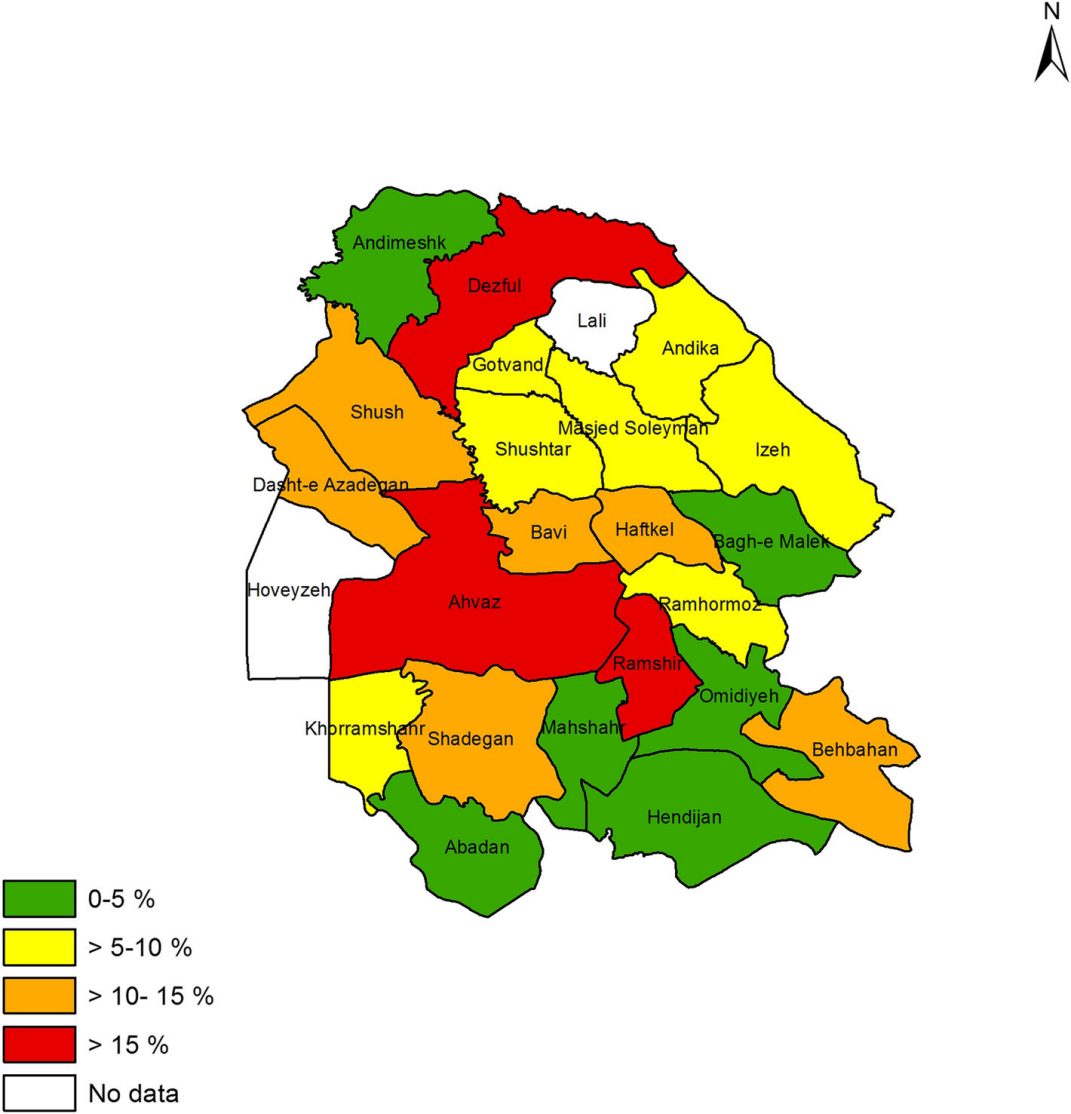
Prevalence of eczema in Khuzestan province

Figures 1 to 4 shows the prevalence of current asthma and other allergic conditions in different counties of Khuzestan Province. As regards the prevalence of current asthma, Ahvaz, Dasht-e Azadegan, Khorramshahr, Dezful, Gotvand and Bagh-e Malek, reported the highest prevalence rate (Fig. 1). With regard to the prevalence of AR, the counties of Shush, Dasht-e Azadegan, Masjed Soleyman and Ramshir were the most prevalent (Fig. 2). Also, among different counties, Ahvaz, Dasht-e Azadegan, Dezful, Masjed Soleyman and Ramhormoz had the highest prevalence of airway hyperresponsiveness (Fig. 3). Finally, the counties of Ahvaz, Dezful and Ramshir, had the highest prevalence of eczema (Fig. 4).

## Discussion

This study was conducted to evaluate the prevalence of asthma, asthma symptoms, AR, eczema and airway hyperresponsiveness in Khuzestan Province as a major industrial and polluted area in Iran and in the Middle East.

The prevalence of current asthma, asthma-like symptoms, AR and airway hyperresponsiveness were estimated to be 8.5, 19.0, 26.8, and 38.7%, respectively. The most common symptoms of asthma were nocturnal cough, chest tightness and wheezing.

A cross-sectional study in 70 countries worldwide found that the global prevalence of physician-confirmed asthma, clinical/treated asthma and adult wheezing were 4.3, 4.5 and 8.6, respectively [27].The prevalence of adult asthma is estimated to be between 1 and 5% in several highly populated, industrialized countries in Asia, including India, China, Singapore, Malaysia, Nepal, Pakistan and Bangladesh [28–30]. It seems that the prevalence of adult asthma in Khuzestan province is higher than the reported prevalence in studies in the several Asian countries.

Another study in adults aged 20–40 years in Riyadh (Saudi Arabia) using the ECRHS questionnaire showed that the prevalence of asthma confirmed by a physician was 11.3%, the prevalence of wheezing in the last 12 months was 18.2%, and the prevalence of drug use for asthma was 10.6% [31]. Given the geographical proximity of Khuzestan Province to the Saudi Arabian desert and the exposure of Khuzestan Province and Saudi Arabia to dust storms, the prevalence of asthma and asthma symptoms in Khuzestan Province is approximately the same as in Riyadh, Saudi Arabia. Based on the study of Fazlollah et al. in Iran [9], the outcomes of current asthma (8.5% vs. 4.7%) and asthmatic symptoms (19.0% vs. 8.9%) in Khuzestan Province were almost twice as high as in Iran.

Considering the results of other countries and nation-wide studies in Iran, Khuzestan Province appears to have a high prevalence of asthma and asthma-related symptoms. Increased prevalence of asthma in Khuzestan Province can be due to high allergic load due to vegetation, geographic location and topography, heavy industries including oil and gas, petrochemicals and steel, and proximity to the Arabian Peninsula and the emerging reindeer resources in the neighboring countries, including Iraq which has been causing the escalation and aggravation of the reindeer storms in the province for several years.

The results of this study showed that the prevalence of physician-confirmed asthma is lower than the prevalence of current asthma. The reason for this can be lack of awareness or commitment of all doctors to the diagnostic guide for asthma, which leads to less asthma diagnosis, or the use of a number of doctors from terminologies other than asthma, such as allergies in humans.

The prevalence of current asthma, asthma symptoms and physician-confirmed asthma was not different between men and women, which is consistent with the results of the study of Fazlollahi et al. (2015) [9]. Indeed, endogenous and exogenous sex steroid hormones cause women to develop asthma after adolescence [32]. Despite the persistence of gender discordance in childhood asthma [33], there is no evidence of asthma and gender in adults [9].

In contrast, the prevalence of AR and airway hyperresponsiveness in women was significantly higher than that of men. According to the results of other studies, the airway hyperresponsiveness of children in boys is higher. However, it is more severe in girls [34–36]. Most studies on the relationship between age and sex with allergic conditions appear to have been conducted in children, and there is little information in this regard in adults.

The results of this study showed that the prevalence of current asthma, asthma symptoms and physician-confirmed asthma increases with higher age, and in contrast to allergic conditions including AR, the airway hyperresponsiveness and eczema decrease with higher age.

Although the new onset of asthma at any age may occur in adults, higher prevalence of asthma at older ages can be due to exposure to environmental factors. However, allergies and atopies as risk factors for asthma are found in other studies to decrease in higher ages [34].

The prevalence of current asthma, wheezing with shortness of breath, wheezing in the absence of colds, nocturnal cough, asthma attack and medications for asthma were significantly higher in cities than in rural areas. In this regard, the results of our study are in line with the results of two studies by Śliwczyński et al. [37] and Kupryś-Lipińska et al. [38] in Poland.

It seems that the reason for the high prevalence of asthma in the city relative to the village in Khuzestan Province is the existence of numerous industries, including the oil and gas extraction industries, petrochemicals, steel and sugar cane and carbon black in the city.

Among the urban factors potentially responsible for increasing the prevalence of asthma and atopic diseases, air pollution resulting from industry and traffic is often cited [38].

The use of medications for asthma was significantly different for urban citizens and villagers, which probably indicates that more people are aware of the use of prescription drugs, especially respiratory inhalers, and more adherence to prescription drugs.

In this study, the prevalence of current asthma, asthma symptoms, wheezing, nocturnal cough and asthma medication was significantly higher in smokers than non-smokers. Various studies similar to ours have shown an association between cigarette smoking and marijuana with asthma and asthma symptoms [7–9, 39–41].

Among the counties of Khuzestan Province, there was a significant difference in the prevalence of current asthma, AR and airway hyperresponsiveness. It seems that this variety suggests that local factors such as ethnicity, race, genetics and various environmental factors alter the prevalence of these conditions in different locations. The reason for this variation in the prevalence of asthma, AR, and airway hyperresponsiveness between different areas of the province can be a topic for future research.

Finally, given that in most epidemiological studies, information about the main symptoms of asthma are filled in the questionnaire by patients, the method is likely to have strengths and weaknesses. One of their limitations is the problem of patients to remember symptoms, as well as individual’s understanding of the disease. Despite efforts to ensure valid responses to questionnaire items about asthma and its symptoms, there may still be controversy among people. In some cases, a positive answer to a questionnaire item does not necessarily indicate asthma. For example, wheezing in a person does not always denote asthma and is also seen in other diseases. However, with the use of a set of asthma symptoms and the confirmation of asthma by a doctor, it seems that a certain credibility can be obtained for the results.

## Conclusion

The high prevalence of asthma in Khuzestan province can be attributed to the high load of allergies resulting from dust storms and the occurrence of respiratory crises and frequent thunderstorm asthma in recent years. This phenomenon requires more attention and planning of health authorities and policy-makers at the level of primary prevention, including environmental measures to prevent and reduce the burden of asthma in the coming years. Due to the high prevalence of chemoprophylaxis as a medical treatment in people susceptible to asthma (rhinitis, increased airway susceptibility), attention can also be paid to preventing asthma, although the results are not conclusive. In sum, surveillance studies are necessary to monitor the trends of the prevalence of asthma in this province.

## Abbreviations

AR: Allergic Rhinitis
DALYs: Disability Adjusted Life Years
ECRHS: European Community Respiratory Health Survey
NO_2_: Nitrogen Dioxide
O_3_: Ozone
SO_2_: Sulfur Dioxide
YLD: Years of Life Lost due to premature death
YLL: Years of Life lived with Disability
